# Chromatin state distribution of residue-specific histone acetylation in early myoblast differentiation

**DOI:** 10.1186/s40537-022-00667-3

**Published:** 2022-12-09

**Authors:** Yuan Li, Saadia Khilji, Yan Z. Mach, Jihong Chen, Qiao Li

**Affiliations:** 1 Department of Cellular and Molecular Medicine, Faculty of Medicine, University of Ottawa, Ottawa, ON Canada; 2 Department of Pathology and Laboratory Medicine, Faculty of Medicine, University of Ottawa, Ottawa, ON Canada

**Keywords:** Epigenetics, Gene regulation, Histone acetyltransferase, Histone acetylation, Stem cell differentiation, Myogenesis

## Abstract

Dynamic changes in epigenetic landscape reflect a critical command of lineage-specific gene expression. In an effort to discern the epigenetic regulatory networks of myogenic differentiation, we have used systematic and integrative approaches to explore multi-omics datasets on global myogenic gene expression, histone acetylation and acetyltransferase occupancy in view of distinct chromatin states. In this brief report, we discuss experimental design and provide a comprehensive assessment regarding data quality control, filtering and processing. We also define a gene-level overlap between RNA-seq and ChIP-seq datasets through integrative analyses to offer strategies for future use of the data. Furthermore, our analyses generate a blueprint on chromatin state distribution of residue-specific histone acetylation and concomitant association with histone acetyltransferase p300 in committed skeletal myoblasts and differential histone acetylation signatures at the onset of myoblast differentiation. These datasets can be further utilized to delineate the function of muscle-specific regulatory elements governed by other muscle myogenic regulators or signaling molecules.

## Background

Epigenetic regulatory mechanisms affect global gene expression patterns underpinning lineage commitment and stem cell differentiation, and thus impact tissue homeostasis including skeletal muscle [1–4]. Recent advances in large-scale functional genomics permit the use of omic approaches to delineate the regulatory roles of epigenetic modifiers in cell fate decision [5]. As such, muscle-specific enhancers and several histone acetylation marks have been identified in terminally differentiated myotubes [4, 6]. We have also conducted ChIP-seq of different histone acetylation marks and histone acetyltransferase (HAT) p300 with condition matching RNA-seq in differentiating and proliferating myoblasts to examine the coupling of epigenetic landscapes with myogenic transcriptome in early myoblast differentiation [7–10].

Different regulatory elements are marked by distinct patterns of chromatin modifications, in that H3K9ac, H3K18ac, and H3K27ac are often found near transcription start sites (TSS), while H4K8ac is mostly associated with promoters and gene bodies that are actively transcribed [11–13]. In addition, H3K4me1 together with H3K27ac are generally considered as marks of active enhancers, while H3K4me1 alone or in accompany of H3K27me3 indicates a poised enhancer [14, 15]. Besides histone modifications, enhancers are also marked by the occupancy of p300 which is a transcriptional co-activator containing an intrinsic HAT activity [16, 17]. While p300 associates with both promoters and enhancers [8, 9, 18], it is well recognized as a signature of enhancers, with H3K18 and H3K27 as main acetylation targets [19–21].

Partaking in transcription control of a vast number of genes, p300 is specifically required for the function of muscle-specific transcription factors and essential for myogenic differentiation [22, 23]. Underscoring a critical role of p300 in myogenic fate decision, embryonic stem cells derived from p300 knockout mice do not express muscle regulatory factors MyoD and Myf5 and knockdown of p300 impedes myogenic differentiation [24, 25].

Cell type specific chromatin states can be defined by large-scale analyses of co-occurrence of different histone marks [26, 27]. We have previously established a 14-state chromatin model based on a genome-wide co-occurrence of various histone modifications to study the regulatory mechanisms of myogenic differentiation [7]. To this end, we used differentiating myoblasts with proliferating myoblasts as control to obtain H4K8ac, H3K9ac, H3K18ac, H3K27ac and p300 ChIP-seq datasets for the survey of global epigenetic landscapes in early myoblast differentiation. These ChIP-seq datasets were generated in matching conditions of our RNA-seq to allow the coupling of epigenetic landscape with global gene expression profiles. Through comprehensive analyses of the omic datasets with an integration of myoblast chromatin states, we have determined the association of p300 and muscle master regulator MyoD with myogenic enhancers and defined a loci-specific enrichment of H4K8 and H3K9 acetylation [8]. In addition, we have also identified the chromatin state distribution of myogenin regulatory loci [9]. Our analyses have established that residue-specific histone acetylation augments at distinctive regulatory elements albeit a global decrease in histone acetylation at the onset of myoblast differentiation.

Since epigenetic modifiers and transcriptional regulators are essential for myogenic differentiation and their dysfunction is implicated in pathogenesis of skeletal muscles, a better grasp of epigenetic regulatory mechanisms of the myoblast genome is paramount. In this report, we provide technical validation and comprehensive analyses of total of 14 genomic datasets which profile global gene expression, histone acetylation, and p300 occupancy for an unbiased and genome-wide view of chromatin states associated with myoblast differentiation. For an in-depth usage of the datasets, we provide detailed information on the experimental design, data quality control, and integrative analyses. Our objective is to offer strategies for future integrative analyses of the datasets to advance the knowledge of epigenetic regulatory networks on myoblast genome in a specific signaling pathway or pathological context. Moreover, comprehensive analyses of epigenomes and transcriptomes in differentiating myoblasts also provide a platform to identify additional histone acetylation signatures of myoblasts genome.

### RNA-seq data processing

Total RNA was extracted from C2C12 myoblasts using RNeasy kit (Qiagen) according to the manufacturer’s instruction. RNA samples from two biological replicates were processed by the McGill University Genome Quebec Innovation Centre for cDNA library construction and deep sequencing using an Illumina HiSeq 2000 sequencer as single-end 50 base pair reads as previously described [7]. Salmon was used to perform mapping-based estimation of transcript abundance guided by transcripts from the NCBIM37.67 database. Gene level counts were subsequently obtained through the tximport package which estimates the counts and transcript lengths for downstream gene-level analysis.

### ChIP-seq data processing

ChIP-seq library preparation and deep sequencing were performed by the McGill University Genome Quebec Innovation Centre with Illumina HiSeq 2000 as single-end 50 bp reads [7]. The sequencing reads were aligned to the mm9 reference genome using Bowtie2, with subsequent removal of duplicate reads by Picard tools. Bedgraph files were created following read extension by 125 bp at the 3’ end with the BEDTools suite. Autocorrelation analysis was used to estimate the fragment length in base pairs. Hypergeometric Optimization of Motif EnRichment (HOMER) was used to call peaks with options -region and -style factor for p300 or -style histone for histone acetylation marks.

The resulting peak files were used as input into HOMER for gene annotation. ChIP enrichment and p300-associated sites was calculated using ngs.plot, which calculates the coverage vectors for each region based on specified ChIP-seq alignment files. Following normalization and transformation on the coverage, a heatmap as well as an average profile is plotted from the mean of all regions, normalized to the total number of mapped reads in millions. Integrative Genomics Viewer (IGV) was used for visualization of the ChIP-seq read signals. Model-based Analysis of ChIP-seq (MACS) was used to determine the scores of Fraction of Reads in Peaks (FRiP) and to call broad peaks of histone acetylation for chromatin states distribution.

### Overview of experimental design

We examined myogenic expression and histone acetylation profiles on a genome-wide scale using C2C12 skeletal myoblasts as a system of differentiation. These myoblasts are myogenic precursors of mesenchymal origin and represent a well-established model of myogenic differentiation. They switch from proliferation to differentiation under low mitogen conditions, followed by a fusion process to form multinucleated myotubes and have been a model of choice for many genome-wide studies due to a high correlation with primary myoblasts [28, 29]. They are also less prone to spontaneous differentiation than primary myoblasts, and thus offer a more homogenous population for the large-scale studies [4, 30].

**Fig. 1:**
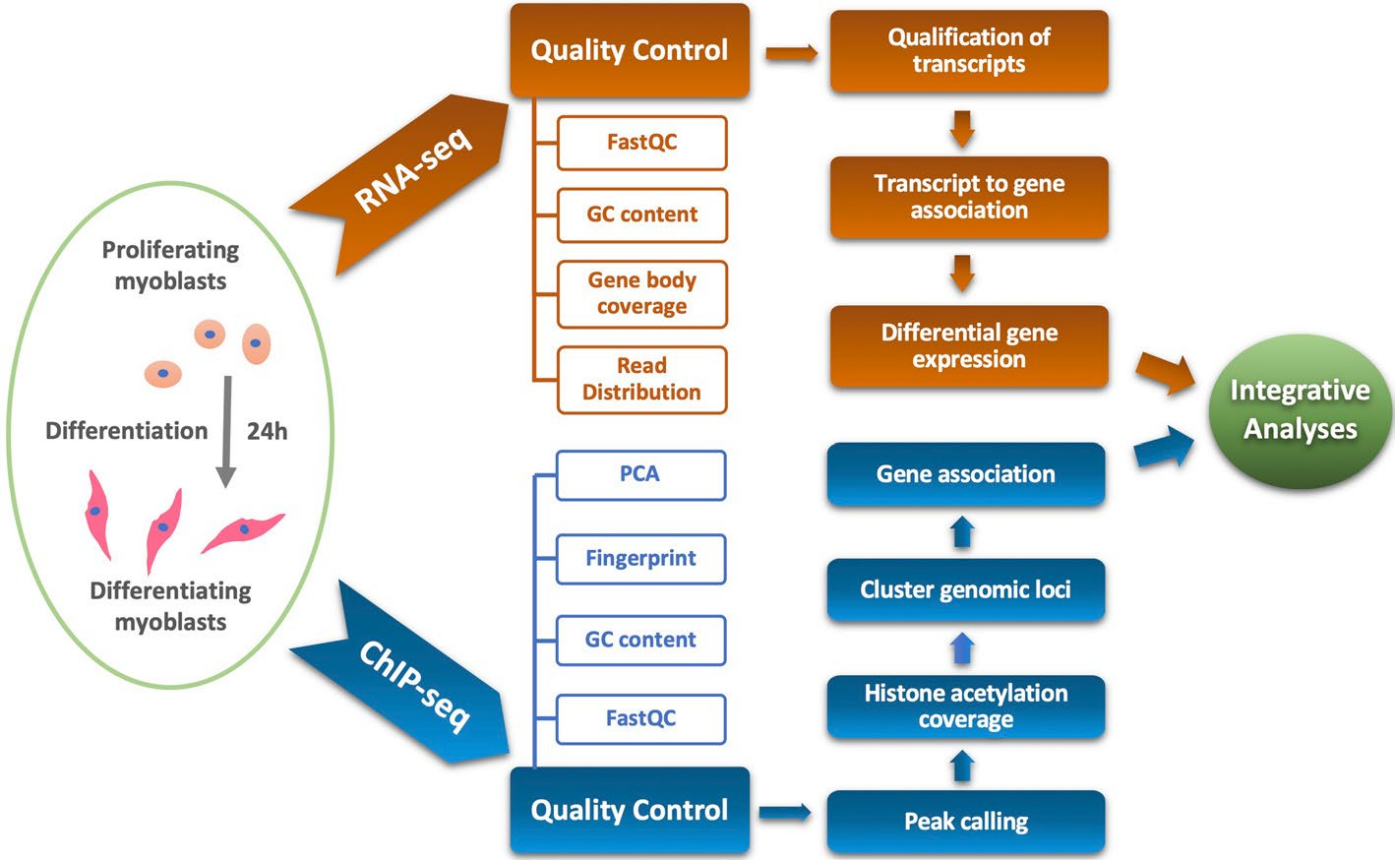
Overview of experimental design and computational analysis workflow. The diagram displays the procedures used for RNA-seq and ChIP-seq analyses of C2C12 myoblasts differentiated for 24 h with proliferating myoblasts as controls. The analyses include data quality control and filtering, differential enrichment or expression analyses, and integration of gene expression with localized ChIP enrichment

In our studies, the C2C12 myoblasts were differentiated for 24 h (Fig. 1) to capture early transcriptional dynamics, since thousands of genes are differentially expressed within 24 h of differentiation [7]. Additionally, our analyses focused on epigenetic changes at the onset of differentiation compared to other studies on terminally differentiated myotubes [4]. Since histone acetylation is known to be transient [31, 32], an earlier time point permits the coupling of epigenetic modifiers with differential gene expression to reveal regulatory networks critical for the initiation of myoblast differentiation. As such, we performed ChIP-seq of H4K8ac, H3K9ac, H3K18ac, H3K27ac and the HAT p300 in matching condition of RNA-seq to capture epigenetic changes of the myoblast genome (Fig. 1). Future analyses of these datasets in view of other signaling pathways or transcription factor association should allow new biological insights into the epigenetic dynamics of myogenic differentiation. Here, we provide the characterization of the datasets with respect to general signal quality, genome alignment, ChIP enrichment, replicate correlation, and integrative analyses.

### RNA-seq quality control

**Fig. 2:**
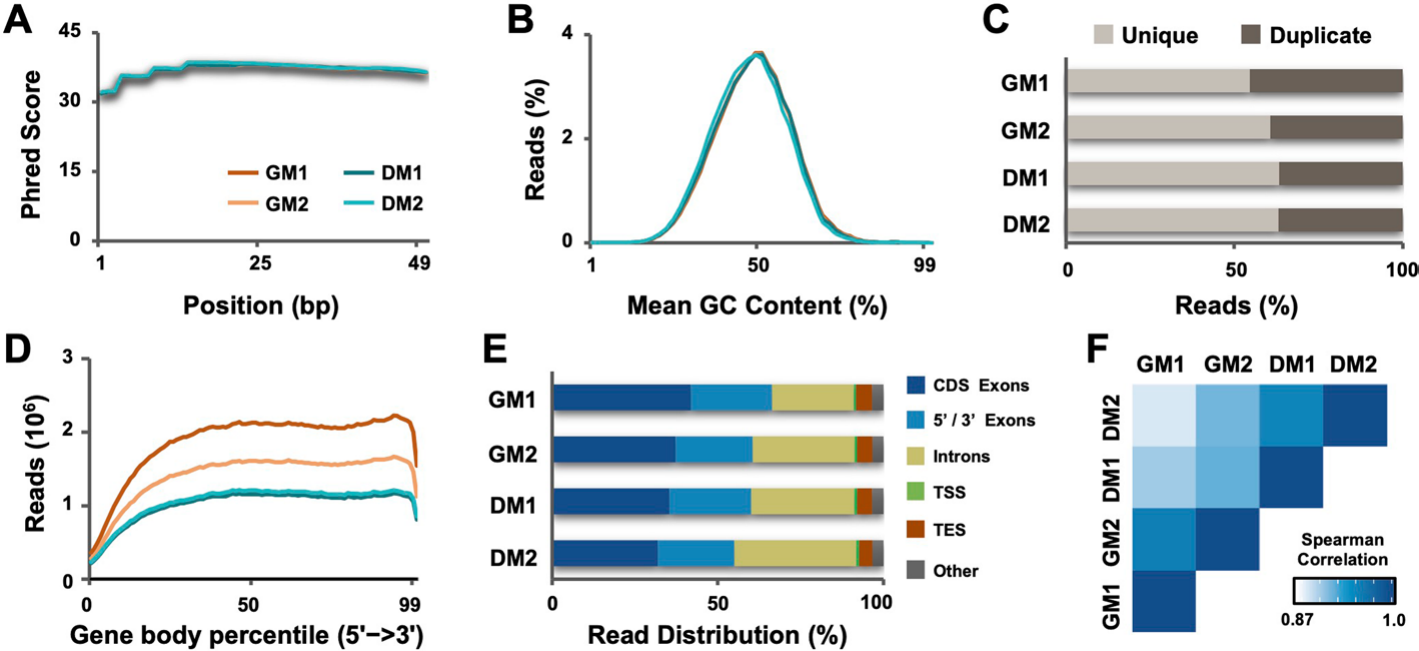
Quality control of the RNA-seq datasets. **A** distribution of base quality scores is shown for two biological replicates of RNA-seq of C2C12 myoblasts following 24 h of differentiation (DM1, DM2) with proliferating myoblasts as controls (GM1, GM2). **B** The distribution of GC content across the reads. **C** The percentage of unique and duplicate reads from all mapped reads in each dataset. **D** The distribution of read coverage across the gene body. **E** The alignment of reads to known gene features in the reference genome (CDS, coding sequence; TSS, transcription start site; TES, transcription end site). **F** Heatmap of Spearman correlation coefficients calculated between log_2_- transformed, nonzero FPKM expression values in both replicates of proliferating and differentiating myoblasts

To assess the quality of RNA-seq datasets for differentiating and proliferating myoblasts, base call scores were analyzed using the FastQC program (Fig. 2A). The quality scores across the reads for all data sets fell within the high confidence range (base quality score of 30–40). Moreover, an inspection of the average GC content across the reads revealed an overall normal distribution representative of a standard random library (Fig. 2B). We also assessed the percentage of unique and duplicate reads across all datasets (Fig. 2C). Theoretically, the number of reads mapping to a certain transcript reflect the number of molecules of the transcript in the sample and thus duplicate reads in RNA-seq can represent highly-expressed genes where deduplication of samples can lead to bias in true expression measurements [33]. As shown in Fig. 2C, the average duplication rate across all samples was approximately 40%, below the normal duplication rate cut-off of 50%, indicating sufficient starting material and library complexity.

Next, the RSeQC gene body coverage tool was used to examine uniformity of read coverage across the gene body for indications of any 5ʹ/3ʹ biases. Figure 2D exhibits relatively even coverage of the reads across the gene body with approximately equal percentage of reads that map near the 5ʹ and 3ʹ end. Previous studies have demonstrated an increased 3ʹ mapping bias with decreasing RNA integrity [34, 35]. Thus, a uniform gene body coverage in all samples is one indicator of suitable quality of the starting RNA material. Interestingly, despite a decrease in the number of reads in differentiating myoblasts compared to proliferating myoblasts, we observed even gene coverage across all conditions (Fig. 2D).

In RNA-seq, distribution of the reads across known gene features is an additional important measure of experimental validity. Most reads are expected to map to exons compared to introns and intergenic regions [36]. As shown in Fig. 2E and Table 1, the exonic rate across all samples was similar at about 60% with an intronic rate of about 35%. The relatively high number of intronic reads are likely a result of the library construction method of using random priming rather than poly-A selection [36]. Nonetheless, the majority of reads mapped to exonic regions, warranting for applications of differential expression analyses.

**Table 1:** RNA-seq quality metrics

RNA-seq sample	SRA ID	Mapped reads	Mapping rate (%)	Exonic rate (%)	Intronic rate (%)	Intergenic rate (%)	rRNA rate (%)	GEO accession number
GM1	SRX2540692	37,330,414	96.3	66.68	28.8	4.6	1.9	GSM2478318
GM2	SRX2540693	32,115,371	96.2	60.2	35.2	4.6	1.6	GSM2478319
DM1	SRX2540698	25,537,075	96.2	59.8	35.5	4.7	1.5	GSM2478324
DM2	SRX2540699	30,280,640	96.1	54.2	41.3	4.6	1.0	GSM2478325

In addition to detecting novel transcripts, a principal benefit of RNA-sequencing has been replicability between samples, which was measured as the correlation of gene expression between samples (Fig. 2F). Spearman correlation analysis revealed greater similarity between replicates for both differentiating and proliferating myoblasts than across conditions. Additionally, the correlation between replicates was approximately 0.95, indicating highly satisfactory technical reproducibility. Overall, our RNA-seq datasets are good resources to guide the validation and functional characterization of early myogenic gene expression and to identify regulatory networks associated with specific signaling pathways.

### ChIP-seq quality control

**Fig. 3:**
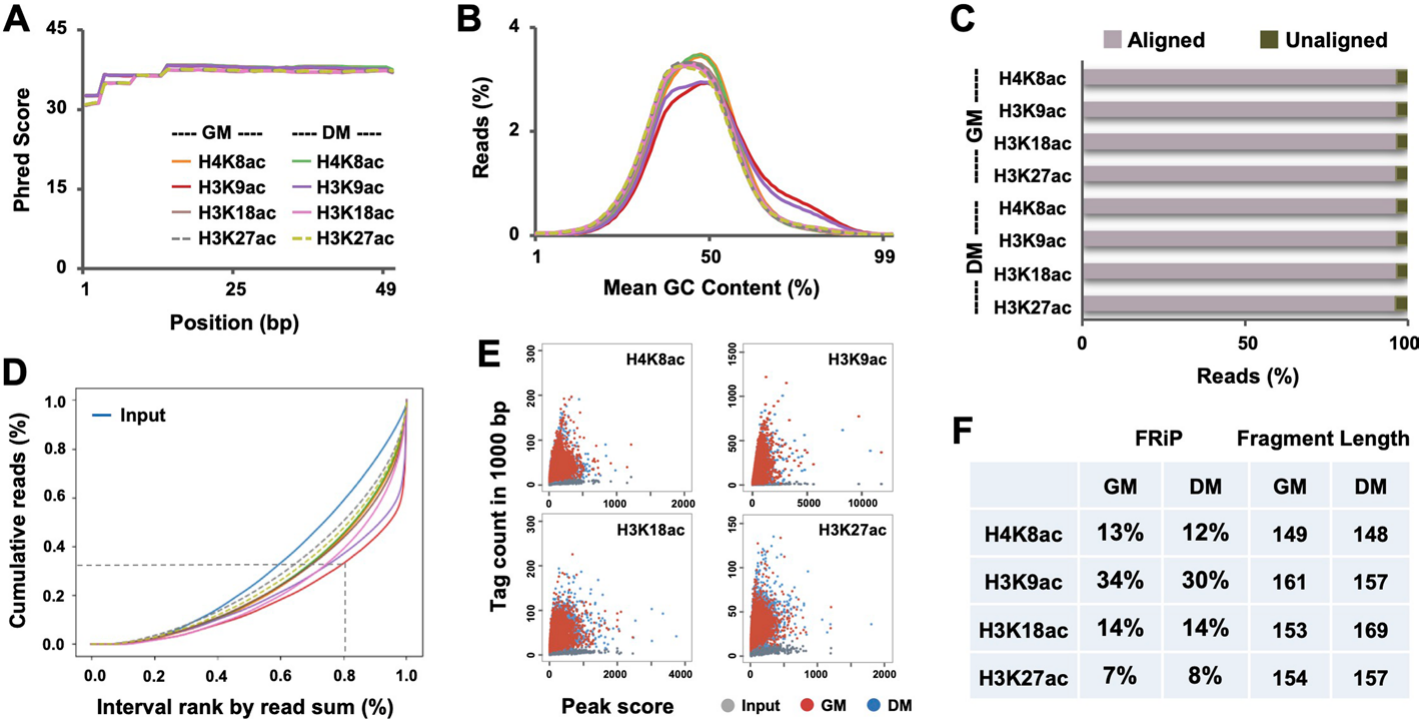
Quality control of the ChIP-seq datasets. **A** The distribution of base quality scores for ChIP-seq of the indicated histone acetylation marks in C2C12 myoblasts differentiated for 24 h (DM) with proliferating myoblasts as control (GM). **B** The average GC content of the read signals. **C** The percentage of reads mapped to the reference genome for each of the histone acetylation marks. **D** Fingerprint plot presents the cumulative percentage of total reads found in a given percentage of the mappable reference genome. **E** Scatter plots of ChIP-seq. enrichments. **F** Shown are the FRiP scores determined by MACS2 and the fragment length in base pairs estimated by autocorrelation analysis

The average base quality scores for all ChIP-seq datasets in the present study fell within the high confidence range (Fig. 3A) and displayed an overall normal distribution for the average GC content of reads (Fig. 3B). Furthermore, raw sequence reads were aligned to the mm9 build of the mouse genome with an average mapping rate of 96% (Fig. 3C), representing an average of 36 million total aligned reads across the samples (Table 2). Subsequent ChIP-seq analysis was performed with uniquely aligned reads to eliminate the obscurity of reads that align to multiple locations. An average of 30 million uniquely aligned reads were retained across all ChIP-seq samples, displaying a mean unique alignment rate of 83% (Table 2).

**Table 2:** Sequencing reads alignment statistics for ChIP-seq datasets

ChIP-seq target	Antibody	Condition	SRA ID	Total mapped reads	Uniquely mapped reads	Unique mapping rate (%)	GEO accession number
p300	sc-584 (Santa Cruz)	GM	SRX3599537	47,518,797	36,874,586	77.6	GSM2947737
		DM	SRX3599538	42,017,222	31,849,054	75.8	GSM2947738
H4K8ac	ab15823 (Abcam)	GM	SRX2540676	34,321,318	29,825,225	86.9	GSM2478290
		DM	SRX2540677	34,787,095	29,986,476	86.2	GSM2478291
H3K9ac	ab4441 (Abcam)	GM	SRX2540679	31,129,778	24,748,174	79.5	GSM2478293
		DM	SRX2540680	38,841,418	30,684,720	79.0	GSM2478294
H3K18ac	ab1191 (Abcam)	GM	SRX2540682	36,763,380	32,131,194	87.4	GSM2478296
		DM	SRX2540683	27,544,482	24,239,144	88.0	GSM2478297
H3K27ac	ab4729 (Abcam)	GM	SRX2540685	35,542,983	30,815,766	86.7	GSM2478299
		DM	SRX2540686	30,247,017	26,375,399	87.2	GSM2478300

Following the confirmation of satisfactory alignment metrics, we also examined the genome coverage and the strength of ChIP-seq simultaneously through cumulative enrichment analysis (Fig. 3D). Intersection of the x-axis at approximately 0.1 by the cumulative distribution of all histone acetylation marks indicates a lack of read coverage for approximately 10% of the mappable genome. However, all datasets exhibited ChIP enrichment in comparison to input, signified by the rightward deflection of a trace which denotes the extent of ChIP enrichment. As an example, following the trace for H3K9ac, the intersection of the dashed lines indicates that 20% of the genome is enriched with about 70% of all uniquely aligned reads for H3K9ac, demonstrating a strong enrichment in the ChIP. Comparable strength of ChIP enrichments and similar pattern of read coverage were evident for other histone acetylation marks as well, shown by their respective cumulative enrichment trace (Fig. 3D). Scatter plot analysis further corroborated the strength of ChIP enrichments as compared to the input control (Fig. 3E). The scores of Fraction of Reads in Peaks (FRiP), ranging from 7 to 34%, all surpassed the 1% benchmark measure by the ENCODE [37] for a successful ChIP experiment (Fig. 3F). Fragment length estimated by autocorrelation analysis for the ChIP-seq was all close to 160 bp, again indicative of successful chromatin fragmentation in the ChIP-seq experiments (Fig. 3F).

### Chromatin state model

We have previously established a model of 14-chromatin states in committed skeletal myoblasts [7], with multiple genome-wide marks of histone acetylation, histone methylation and RNA-Pol II, by using a hidden Markov model-based method [26]. These chromatin states are categorized as promoters, enhancers, and “others” that include genomic regions which are either actively transcribed, or inactive/repressed. The enhancer states are classified by the presence of H3K4me1 but absence of H3K4me3 and H3K36me3, and further divided into active or poised enhancers according to histone acetylation signal intensity. The promoter states are characterized by the enrichments of H3K4me3, transcription start sites (TSSs) and RNA Pol II occupancy, and subdivided into active or poised promoters based on the levels of histone acetylation. This chromatin state model was employed in our studies to index p300-associated histone acetylation sites into distinct chromatin states to discern the epigenetic regulatory networks of myogenic differentiation [7–10].

### Chromatin states associated with residue-specific histone acetylation

**Fig. 4:**
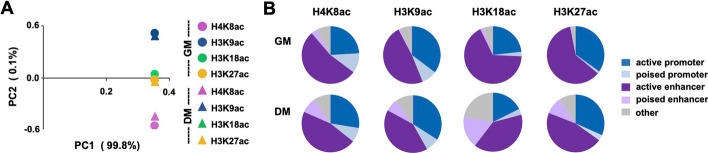
Chromatin state associated with residue-specific histone acetylation. **A** C2C12 myoblasts were differentiated for 24 h (DM) and subjected to ChIP-seq with proliferating myoblasts (GM) as control. Shown is the PCA plot for the indicated histone acetylation read signals. **B** The distribution of H4K8ac, H3K9ac, H3K18ac, and H3K27ac read signals to distinct chromatin states

Here, we analyzed ChIP-seq of histone acetylation marks including H4K8ac, H3K9ac, H3K18ac, and H3K27ac after quality verification of the raw sequencing data, since transcriptomes are controlled by epigenetic regulatory networks. PCA plots were first used to assess the clustering of histone acetylation marks into their respective biological groups for chromatin modification and differentiation status (Fig. 4A). The plot revealed close clustering of H3K18ac and H3K27ac signals in comparison to H4K8ac and H3K9ac which were distinct from one another. Since H3K18ac and H3K27ac enrichment in addition to p300 occupancy are key features that denote enhancers [15, 20], the close correlation of these marks suggests a co-regulation of myogenic enhancers during the differentiation process. Furthermore, the inter-modification differences were greater than the variation between proliferation and differentiation myoblasts as expected.

Next, we examined the chromatin state distribution of residue-specific histone acetylation in early differentiation utilizing our established chromatin state model. As shown in Fig. 4B, H3K18ac and H3K27ac had a more pronounced association with the enhancers (69% and 61%, respectively) in proliferating myoblasts, whereas H4K8ac and H3K9ac displayed a clear association with poised promoters (11% and 9%, respectively). Most interestingly, there was an evident increase in the distribution of histone acetylation marks to poised enhancers in differentiating myoblasts when compared to proliferating myoblasts, with the largest increase found in H3K18ac and H3K27ac signals, to 16% and 9%, respectively (Fig. 4B). Nonetheless, the distribution of H4K8ac and H3K9ac to the promoters were similar in both proliferating and differentiating myoblasts (Fig. 4B). Established in committed myoblasts, poised enhancers are marked by discrete histone modification and critical for governing gene programs required for myoblast differentiation and development [38]. Thus, an increased distribution of residue-specific histone acetylation to poised enhancers reflects their involvement in the activation of myogenic programs.

### Residue-specific histone acetylation at p300-associated sites in early myoblast differentiation

**Fig. 5:**
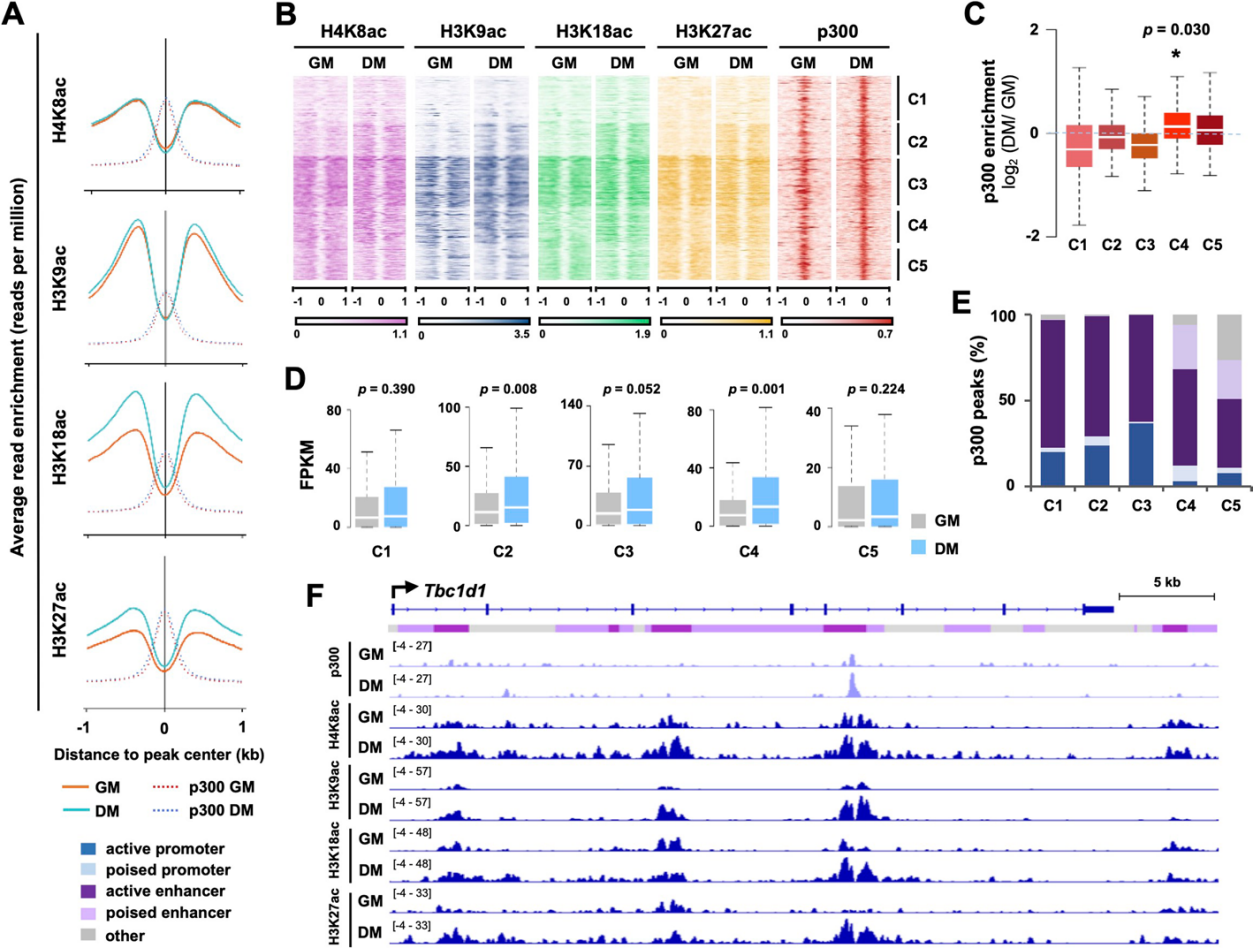
Histone acetylation associated with p300 peaks in early myoblast differentiation. **A** C2C12 myoblasts were differentiated for 24 h (DM) and subjected to ChIP-seq with proliferating myoblasts (GM) as control. Shown are the profiles of average ChIP-seq read signals for the indicated histone acetylation marks at the sites of p300 occupancy. **B** The p300 sites in differentiating myoblasts were used to categorize enriched histone acetylation signals into 5 clusters with k-means clustering. The signal density is presented ± 1 kb from the peak canter. **C** Quantification of p300 enrichment signals was plotted as log_2_-fold change in differentiating myoblasts relative to proliferating myoblasts. **D** Gene expression levels from the RNA-seq are presented as FPKM (Fragments Per Kilobase of transcript per Million mapped reads) for genes associated to the indicated p300 cluster. **E** The p300 sites in each cluster were classified into distinct chromatin states. **F** Genome browser snapshot displays the read density of p300 and histone acetylation at the *Tbc1d1* locus. Blue bars show the Refseq gene position and the colours of ChromHMM track below correspond to that assigned to each chromatin state

We also delineated epigenetic networks involved in p300-mediated gene expression, since p300 is a critical HAT in myogenic differentiation [23, 24, 39]. We first examined the residue-specific histone acetylation profile associated with p300 sites at large (Fig. 5A). Interestingly, p300-associated sites were generally coupled with an enrichment of H3K18ac and H3K27ac in differentiating myoblasts as compared to proliferating myoblasts (Fig. 5A). Next, we correlated the levels of histone acetylation to 5 distinct clusters centered at the p300 peaks in differentiation myoblasts, using k-means clustering (Fig. 5B). An increase in histone acetylation signals during the transition from proliferation to differentiation was parallel to a significant increase in p300 read signals in cluster 4 (Fig. 5B, C). Similarly, the expression of gene associated with this cluster were significantly increased in differentiating myoblasts compared to proliferating myoblasts as determined by the RNA-seq analysis (Fig. 5D). Interestingly, the p300 clusters also exhibited a distinct pattern of chromatin state distribution where p300 peaks in cluster 4 and 5 were clearly associated with the poised enhancers (Fig. 5E). Moreover, the correlation of increased histone acetylation levels with parallel p300 enrichments in differentiating myoblasts is well exhibited by an IGV snapshot (Fig. 5F) at the putative regulatory locus of *Tbc1d1,* a glucose regulator in skeletal muscle [40].

## Conclusion

Integrative analyses of p300 and histone acetylation ChIP-seq data with corresponding chromatin state and transcriptome offer an invaluable avenue to identify chromatin states distribution of residue-specific histone acetylation and discern the distinct function of HATs in epigenetic landscape and gene regulatory networks within a specific biological milieu. The high throughput sequencing datasets presented here are biologically validated and in high quality, and thus can be used for future studies to determine the roles of muscle regulatory factors, residue-specific histone acetylation and p300 function in early myogenic differentiation, particularly in terms of their impact on chromatin dynamics.

## Data Availability

RNA-seq FPKM_tracking files as well as ChIP-seq bedgraph files for histone acetylation marks have been deposited in the NCBI Gene Expression Omnibus (GEO) SuperSeries under accession number GSE94561. Bedgraph and peak files for p300 have been deposited under accession number GSE109636. GEO linked FASTQ and bam files were also deposited in the Sequence Read Archive (SRA). Tables 1 and 2 contain the associated database identification numbers for each dataset.
